# A Successful Pregnancy with Amyotrophic Lateral Sclerosis

**DOI:** 10.1155/2020/1247178

**Published:** 2020-02-29

**Authors:** P. D. M. Pathiraja, S. K. Ranaraja

**Affiliations:** ^1^Obstetrics and Gynaecology, Ministry of Health, Sri Lanka; ^2^Teaching Hospital Peradeniya, Sri Lanka

## Abstract

Amyotrophic lateral sclerosis (ALS) is a degenerative motor neurone disease that is rarely seen in the obstetric population. We present a 32-year-old patient who presented in her fourth pregnancy with a background history of ALS. There was complete involvement of the upper and lower motor neurone system and bulbar system without involvement of the sensory system. At 34 weeks of gestation, she had a full course of steroids and an elective caesarian section under general anaesthesia due to extreme restricted mobility and difficulty of breathing. A multidisciplinary team managed the pregnancy, and both maternal and fetal outcomes were good.

## 1. Introduction

Amyotrophic lateral sclerosis is the most common and progressive motor neurone disease, and the prevalence is 2-4/100,000 in general population [1, 2]. The main problem during pregnancy is respiratory compromise. The mode of delivery and timing of delivery would be affected by the severity of the disease and other comorbid factors.

## 2. Case Report

A thirty-two-year-old lady in her fourth pregnancy came for her booking visit at ten weeks of gestation with a known history ALS for the last two years. Her obstetrics history included one previous vaginal delivery fifteen years prior, an emergency lower uterine segment caesarian section (LSCS) nine years prior for fetal distress, and two first trimester miscarriages in between these pregnancies. Four years following her emergency LSCS, she developed bilateral lower limb weakness and slurring of speech. Neurological examination showed generalized weakness with quadraparesis.

Routine investigations at presentation revealed a normal full blood count, serum biochemistry, serum vitamin B12, serum folate level, ferritin levels, and coagulation profile. Inflammatory markers such as ESR and CRP were normal. She had a negative rheumatoid factor, ANA, and ANCA. She had undergone an electromyelogram (EMG), which confirmed the presence of widespread anterior horn cell dysfunction consistent with ALS. Magnetic resonance imaging (MRI) of the spine revealed degenerative changes at C5/C6 and C6/C7 levels.

The current pregnancy was unplanned and her main problems during pregnancy were restricted mobility and shortness of breath. At booking, she was bed bound and reported generalized body weakness. On general examination, she had slurred speech, drooling of saliva, and no evidence of pallor or cyanosis. Her cardiovascular examination showed a heart rate of 76 beats per minute, blood pressure was 120/70 mmHg, and there were no audible murmurs. Her respiratory system examination revealed that respiratory rate of 26 breaths per minute with the use of accessory muscles. There was obvious intercostal and subcostal muscle recession during breathing with reduced air entry on both sides. On neurological assessment, both upper and lower limbs were wasted and fasciculation were noted. Assessment of power in all four limbs revealed 3/5, increased tone, with significant spasticity, and hyperreflexia. Her sensory function was unimpaired. Cranial nerve examination showed wasting of tongue muscles.

During the antenatal period, she was referred to a cardiologist and echocardiography was normal. She underwent serial lung function tests, which showed a severe restrictive type lung disease. With the help of a dietician and nutritionist, she continued with a semisolid diet. She had stockings and limb physiotherapy for prevention of deep vein thrombosis. The trained nursing staff regularly assessed her bowel and urinary habits while she is at home. She had muscle relaxant medication to relieve of her spasticity. Fetal anomaly ultrasound scan was normal and fetal growth was satisfactory. She was admitted to the ward at thirty weeks of gestation for close monitoring and further care.

At thirty-four weeks of gestation, the patient complained of progressive worsening of her shortness of breath. A multidisciplinary team (MDT) was gathered including a consultant obstetrician, neonatologist, anaesthetist, and neurologist to discuss the timing and mode of delivery. After the MDT meeting, a decision was made for an elective LSCS followed by monitoring at intensive care unit. Steroids were given for fetal lung maturity. The LSCS was performed without any complications, and the baby's birth weight was 2.4 kg and the Apgar score, neurological, and other system examination of the neonate was normal. The patient made quite an uneventful recovery with improvement in her lung functions and motor weakness remained stable. Both mother and baby were discharged home seven days post LSCS.

## 3. Discussion

The published literature of patients with ALS complicating pregnancy is very rare. This is partly because the incidence of the condition is highest among the elderly population particularly after the fifth decade. In addition, it is seen more commonly in men than women [1, 2]. The management of motor neurone disease in pregnancy especially ALS is challenging for both the obstetrician and patient. Pregnancy does not impact on with degenerative nature of the disease, however, both upper and lower motor neurone deficits can be seen [3].

Respiration is the main component, which get affected during pregnancy. In pregnancy, there is a 40% increase in minute ventilation, which is achieved exclusively by increased tidal volume. In ALS, diaphragmatic and costal muscles may be involved; thus, patient is unable to increase tidal volume. In the latter part of pregnancy, the diaphragmatic elevation caused by the enlarging uterus leads to a decrease in functional residual capacity. As a result, serial review of respiratory function by pulmonary function tests is recommended especially towards the end of pregnancy [4].

Riluzole is the only proven drug, which is effective in ALS and can be used during pregnancy. Food and Drug Administration (FDA) categorize it as a class C drug. It delays the requirement of ventilator dependency and may increase the duration of survival. The low birth weight has been reported when used during pregnancy [5]. Furthermore, liver functions need to be assessed every three months due to hepatotoxicity.

The mode of delivery in ALS is controversial. In normal pregnancy, the largest increase of cardiovascular and respiratory work occurs after birth and should be similar for either routes of delivery. Since motor neurone diseases do not affect the motor and sensory nerves of the uterus, a vaginal delivery is possible. However, if the patients have respiratory failure, she will need a cesarean section as spontaneous labour increases respiratory demand. Chio et al. report a case series of four patients of whom three had uncomplicated vaginal deliveries [6]. Sarfov et al. report a case of a single women with ALS, who delivered vaginally in her first pregnancy; however, she required a caesarian section in early third trimester due to worsening of the disease condition [7].

Choice of anaesthesia for patients with ALS is complicated. Regional anaesthesia offers the advantage of sustained pain relief and this together with general anaesthesia is associated with similar reductions in lung volume. However, the effects of regional anesthesia on intercostal muscles may impede expiratory flow that is of concern in patients with respiratory impairment. Xiao et al. report successful use of total intravenous short acting anesthesia without muscle relaxant. This would help to avoid any prolonged ventilation, therefore, prevent maternal respiratory compromise [8]. Corderio et al. present a case of noninvasive ventilation and spinal anaesthesia was used in a patient with rapid deterioration of ALS in pregnancy by avoiding intubation and total anaesthesia [9].

Since motor neurone disease does not affect fetal development, neonatal outcomes are generally good. Some case reports suggest an association of fetal growth restriction with Riluzole. There are other case series that have shown an association with congenital anomalies, such as anencephaly, cleft lip, and cleft palate, but this association is unclear [10].

The relationship between the hormonal change in pregnancy and increased susceptibility to ALS has been studied. A study done using patients who attend a neuromuscular clinic found mutation of the superoxide dismutase gene (SOD1) and vascular endothelial growth factor premotor gene (VEGF) could be a causative factor [11]. Both hormonal and inflammatory modification during pregnancy would result in oxidative stress, which leads to mutation of SOD 1 and VEGF molecules, which has toxic mediation and neuroprotective ability. There are some case reports that discuss the onset of ALS in the immediate postpartum period. Martinez et al. report a case of stem cell therapy in a patient who developed ALS during pregnancy. Bilateral autologous stem cell transplantation into the frontal motor cortex has been performed after delivery and disease progression was stabilized with improved quality of life [12]. By considering the long-term impact of the disease and complications could arise during pregnancy, it is advisable that proper counseling of the patient occur preconception.

## 4. Conclusion

ALS in pregnancy is very rare. Pregnancy in women with motor neurone diseases specifically ALS is a potentially dangerous event with respiratory compromise being the main problem encountered. Riluzole can be safely used during the pregnancy. MDT involvement is essential in optimizing care of patients with ALS in pregnancy with expected good maternal and fetal outcome.
